# Is the ability of the Eating Assessment Tool (EAT-10) to screen for aspiration in patients with dysphagia depending on the patients’ disease?

**DOI:** 10.1007/s00405-022-07395-7

**Published:** 2022-05-03

**Authors:** Ole Schlickewei, Julie Cläre Nienstedt, Christina Pflug

**Affiliations:** Department of Voice, Speech and Hearing Disorders, Center for Clinical Neurosciences, University Medical Center Hamburg-Eppendorf, Martinistrasse 52, 20251 Hamburg, Germany

We would like to express our sincere appreciation for the interest of Dr. Printza in our recently published paper and thank the journal for the opportunity to respond to points raised.

We are pleased that our study is the subject of further consideration and investigation.

Printza states that the mean Eat-10 scores of our patient cohort differ significantly from EAT-10 scores in other studies.

We agree that these differences are mainly due to cognitive deficits in Parkinson's disease (PD) [1]. Our studied cohort included 50 patients of whom 19 (38%) showed cognitive deficits in the Montreal cognitive assessment (MOCA) [2]. This means the ability of self-perception and the ability to report may be limited. Thus, the use of subjective self-reporting screening tools may naturally be associated with deficits in predictive function.

In addition, we also draw on a large extent on impairment of laryngopharyngeal sensitivity in Parkinson’s disease to explain the results [3, 4]. Up to 25% of all patients with Parkinson’s disease suffer from silent aspiration [5], which cannot be quantified by the patient himself.

We agree with Dr. Printza that the EAT-10 is a valid screening tool for the evaluation of dysphagia in general but emphasize that there are strong limitations to its use in PD patients. According to the results of our study, we cannot recommend the use of the Eat-10 in PD patients [6].
